# *Lactobacillus casei* Strain Shirota Enhances the In Vitro Antiproliferative Effect of Geniposide in Human Oral Squamous Carcinoma HSC-3 Cells

**DOI:** 10.3390/molecules23051069

**Published:** 2018-05-03

**Authors:** Yu Qian, Jia-Le Song, Peng Sun, Ruokun Yi, Honglin Liu, Xia Feng, Kun-Young Park, Xin Zhao

**Affiliations:** 1 Chongqing Collaborative Innovation Center for Functional Food, Chongqing University of Education, Chongqing 400067, China; qianyubaby@126.com (Y.Q.); sunpeng@foods.ac.cn (P.S.); yirk@cque.edu.cn (R.Y.); aishanglu123@163.com (H.L.); fengxia@foods.ac.cn (X.F.); 2 Chongqing Engineering Research Center of Functional Food, Chongqing University of Education, Chongqing 400067, China; 3 Chongqing Engineering Laboratory for Research and Development of Functional Food, Chongqing University of Education, Chongqing 400067, China; 4 College of Biological and Chemical Engineering, Chongqing University of Education, Chongqing 400067, China; 5 Department of Nutrition and Food Hygiene, School of Public Health, Guilin Medical University, Guilin 541004, China; songjiale@glmc.edu.cn; 6 School of Tourism and Service Management, Chongqing University of Education, Chongqing 400067, China; 7 Department of Food Science and Biotechnology, Cha University, Seongnam 13488, Gyeongghi-do, Korea

**Keywords:** *Lactobacillus casei* strain Shirota, geniposide, human oral squamous carcinoma HSC-3 cells, antiproliferative effect

## Abstract

This study investigated the enhanced antiproliferative effect of *Lactobacillus casei* strain Shirota (LcS) on geniposide actions in human oral squamous carcinoma HSC-3 cells. An MTT assay, flow cytometry, qPCR assay, western blot and HPLC were used for this study. The concentration of 1.0 × 10^6^ CFU/mL of LcS had no effect on the HOK normal oral epithelial cells and HSC-3 cancer cells. The 25 and 50 µg/mL geniposide concentrations also had no impact on HOK normal oral epithelial cells, but they had remarkable inhibitory effects on the growth of HSC-3 cancer cells, which are enhanced in the presence of LcS. By the flow cytometry assay, the LcS-geniposide-H (1.0 × 10^6^ CFU/mL LcS and 50 µg/mL geniposide)-treated HSC-3 cancer cells had the largest number of cells undergoing apoptosis compared to cells treated with other combinationsand obviously more than cells treated with only geniposide-H (50 µg/mL geniposide). Geniposide-H could increase the mRNA and protein expressions of caspase-3, caspase-8, caspase-9, Bax, p53, p21, IκB-α, Fas, FasL, TIMP-1, and TIMP-2 as well as decrease those of Bcl-2, Bcl-xL, HIAP-1, HIAP-2, NF-κB, COX-2, iNOS, MMP-2, and MMP-9 compared to other groups of cells, and LcS further enhanced these changes, with results that are greater than for the cells treated with only a high concentration of geniposide. The results of this study show thatLcS enhanced the antiproliferative effect of geniposide in HSC-3 cancer cells.

## 1. Introduction

*Gardenia jasminoides* Ellis is plant of the *Rubiaceae* family and *Gardenia* genus, and its dried ripe fruit can be used as a medicine [1]. *Gardenia* fruit mainly protects the liver and nourishes the gallbladder, and it contains the active component geniposide, which belongs to the iridoid glycosides [2]. It has many other useful components such as organic acids, pigments and volatile oils [3]. According to a pharmacokinetics study, geniposide is the active constituent of *Gardenia jasminoides* Ellis, which is easily hydrolysed by β-glucosidase, the product of intestinal microorganisms, to generate genipin [4]. Many scientists have results that indicate that genipin has a significant effect in diminishing inflammation, lipid peroxidation and angiogenesis, while having a very low cytotoxicity, sound biocompatibility and high anti-degradation ability [5,6]. *Gardenia jasminoides* Ellis has a very low genipin content, which only accounts for 0.005% to 0.01%, and it also exists in the form of its precursor geniposide, which accounts for 3% to 5%. Currently, geniposide is extracted by an organic solvent, and we can easily extract approximately 4 g of geniposide from 100 g of gardenia fruit. Ultimately, we can understand that the fermentation of the Chinese medicine *Gardenia* by microorganisms to produce genipin has importance [7]. In addition, the use of geniposide together with microorganisms has a remarkable effect on the active constituent to achieve a better effect. The mechanism of transforming *Gardenia jasminoides* Ellis with microorganisms is to ferment geniposide with the bacteria producing β-glucosidase, as β-glucosidase can break the bonds of geniposide to produce genipin [8]. In this study, we used high-yield *lactobacillus* producing β-glucosidase to react with geniposide and observed the joint effects on cancer cells.

Organisms balance the number of tissues and cells by proliferation and apoptosis. When this balance is affected, it can lead to uncontrolled proliferation, and this can lead to cancer and many other different kinds of diseases [9]. The basic relationship between apoptosis and cancer provide a new reference for the treatment of cancer. In the recent 30 to 50 years, experiments on cytotoxic radiotherapy and chemotherapy have become the main treatment measures for cancer, which have certain therapeutic effects towards certain types of haematological malignant tumours, some solid tumours, especially germ cell tumours, and malignant tumours in children to some extent [10]. However, these measures have limited effects on these cells; while high dose chemotherapy can improve the resistance, it cannot cause the apoptosis of all of the cancer cells, and it can also damage normal tissues and cells. From previous studies, it is believed that tumours can be treated by killing the target cells, which are dividing rapidly and selectively, but this is not very helpful in clinical practice, as some treatable cancer cells can still grow, and those with resistance divide rapidly [11]. Some of the studies also focused on the way that treatment may induce the apoptosis of tumour cells, and we know that various cells have different apoptosis thresholds, making their responses to treatment differ. As the induction and regulation of apoptosis are very complex processes, the mechanisms of inducing apoptosis by various tumour drugs are completely different in many aspects [12]. This study observed the significant effects of *Lactobacillus casei* strain Shirota with geniposide on the apoptosis of cancer cells and found the related processes, which helps in providing evidence for the application of *Lactobacillus* combined with geniposide in experimental practices.

## 2. Results

### 2.1. Growth Inhibitory Effects of LcS and Geniposide in HOK and HSC-3 Cells

**Figure 1:**
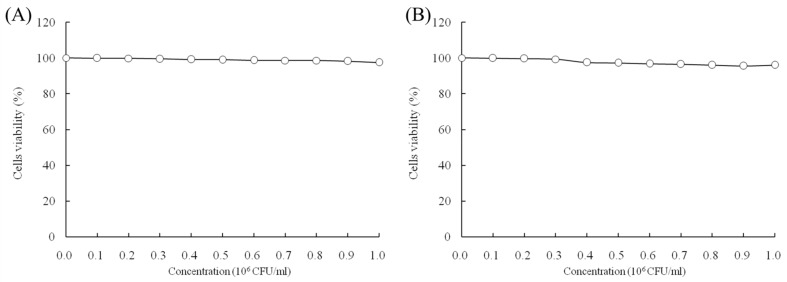
Effects of *Lactobacillus casei* strain Shirota (LcS) (**A**) and geniposide (**B**) on the growth of HOK normal oral epithelial cells.

**Figure 2:**
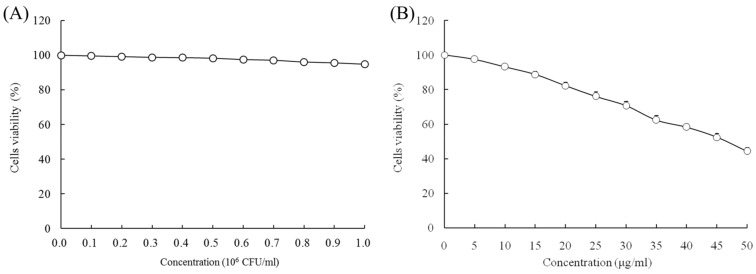
Effects of *Lactobacillus casei* strain Shirota (LcS) (**A**) and geniposide (**B**) on the growth of human oral squamous carcinoma HSC-3 cells.

When the concentrations of LcS and geniposide were 0–10^6^ CFU/mL and 0–50 μg/mL, respectively, they could not prevent the growth of normal HOK cells (Figure 1). With a LcS concentration 0–10^6^ CFU/mL, there was no effect on growth of HSC-3 cancer cells (Figure 2A) by determination of MTT assay. However, a concentration of geniposide of 0–50 μg/mL could inhibit the growth of HSC-3 cancer cells (Figure 2B). Thus, 1.0 × 10^6^ CFU/mL of LcS along with 25 and 50 μg/mL of geniposide were taken as the model treatment for the experiment. Meanwhile, the only LcS (1.0 × 10^6^ CFU/mL) treatment had only a little inhibitory effect (5.2%) in HSC-3 cells (Table 1 and Figure 2B). The 25 and 50 μg/mL geniposide treatments had good inhibitory effects (23.8% and 55.7%) on cancer cells. After the addition of 1.0 × 10^6^ CFU/mL LcS treatment, the geniposide treatment showed better inhibitory effects on cancer cells than only geniposide or LcS treatment (Table 1).

### 2.2. Sub-G1 Content of HSC-3 Cells

By the flow cytometry experiment, the apoptotic cells (sub-G1 DNA content) of the control-, geniposide-L-, LcS-geniposide-L-, geniposide-H- and LcS-geniposide-H-treated HSC-3 cells were 2.4 ± 0.2%, 10.3 ± 0.5%, 16.2 ± 0.4%, 21.8 ± 0.8% and 35.5 ± 1.6%, respectively.

### 2.3. mRNA and Protein Expressions of Caspase-3, Caspase-8 and Caspase-9 in HSC-3 Cells

**Figure 3:**
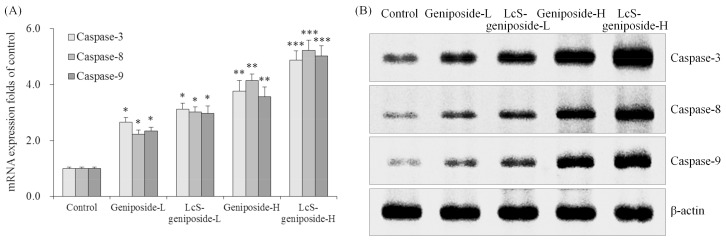
The mRNA (**A**) and protein (**B**) expressions of caspase-3 caspase-8 and caspase-9 in human oral squamous carcinoma HSC-3 cells. * *p* < 0.05, ** *p* < 0.01, *** *p* < 0.001 vs. the control group. Geniposide-L: 25 µg/mL geniposide; LcS-geniposide-L: 1.0 × 10^6^ CFU/mL LcS + 25 µg/mL geniposide; Geniposide-H: 50 µg/mL geniposide; LcS-geniposide-H: 1.0 × 10^6^ CFU/mL LcS + 50 µg/mL geniposide.

As per the results obtained, the geniposide treatment could raise the caspase-3, caspase-8 and caspase-9 mRNA and protein expressions compared to the control cells (Figure 3). Moreover, the higher concentration of geniposide showed higher caspase-3, caspase-8 and caspase-9 expressions. Additionally, LcS could enhance the effects of geniposide treatment on the caspase-3, caspase-8 and caspase-9 expressions.

### 2.4. mRNA and Protein Expressions of Bax, Bcl-2 and Bcl-xL in HSC-3 Cells

**Figure 4:**
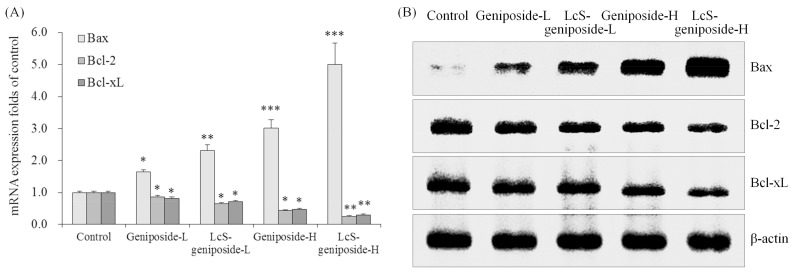
The mRNA (**A**) and protein (**B**) expressions of Bax, Bcl-2 and Bcl-xL in human oral squamous carcinoma HSC-3 cells. * *p* < 0.05, ** *p* < 0.01, *** *p* < 0.001 vs. the control group. Geniposide-L: 25 µg/mL geniposide; LcS-geniposide-L: 1.0 × 10^6^ CFU/mL LcS + 25 µg/mL geniposide; Geniposide-H: 50 µg/mL geniposide; LcS-geniposide-H: 1.0 × 10^6^ CFU/mL LcS + 50 µg/mL geniposide.

After treatment with geniposide-L, LcS-geniposide-L, geniposide-H and LcS-geniposide-H, Bax mRNA and protein expressions increased significantly in turn, but on the contrary, Bcl-2 and Bcl-xL expressions decreased on the same order (Figure 4).

### 2.5. mRNA and Protein Expressions of p53 and p21 in HSC-3 Cells

**Figure 5:**
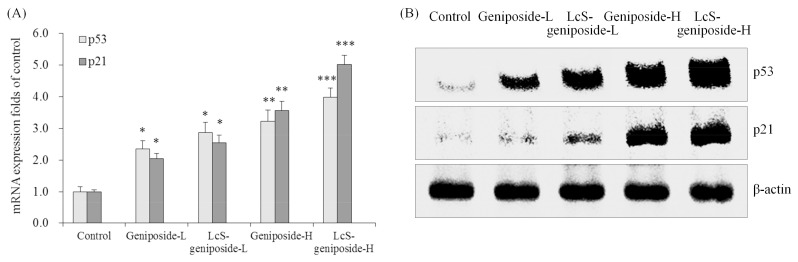
The mRNA (**A**) and protein (**B**) expressions of p53 and p21 in human oral squamous carcinoma HSC-3 cells. * *p* < 0.05, ** *p* < 0.01, *** *p* < 0.001 vs. the control group. Geniposide-L: 25 µg/mL geniposide; LcS-geniposide-L: 1.0 × 10^6^ CFU/mL LcS + 25 µg/mL geniposide; Geniposide-H: 50 µg/mL geniposide; LcS-geniposide-H: 1.0 × 10^6^ CFU/mL LcS + 50 µg/mL geniposide.

The p53 and p21 mRNA and protein expressions of the LcS-geniposide-H group were remarkably stronger than all of the other groups of cells (Figure 5). Additionally, the results showed that the LcS + geniposide combination treatment showed higher p53 and p21 expressions than the geniposide treatment exclusively.

### 2.6. mRNA and Protein Expressions of HIAP-1 and HIAP-2 in HSC-3 Cells

**Figure 6:**
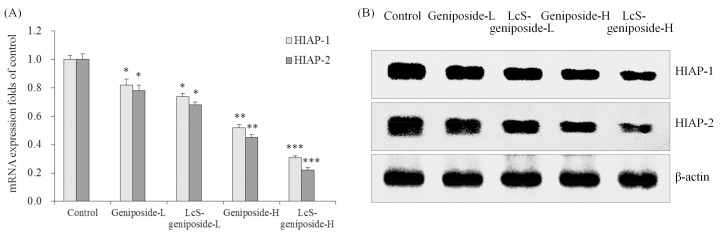
The mRNA (**A**) and protein (**B**) expressions of HIAP-1 and HIAP-2 in human oral squamous carcinoma HSC-3 cells. * *p* < 0.05, ** *p* < 0.01, *** *p* < 0.001 vs. the control group. Geniposide-L: 25 µg/mL geniposide; LcS-geniposide-L: 1.0 × 10^6^ CFU/mL LcS + 25 µg/mL geniposide; Geniposide-H: 50 µg/mL geniposide; LcS-geniposide-H: 1.0 × 10^6^ CFU/mL LcS + 50 µg/mL geniposide.

Geniposide could reduce the mRNA and protein expressions of HIAP-1 and HIAP-2 in HSC-3 cells compared to the control cells (Figure 6), the 50 μg/m geniposide-treated cancer cells had lower HIAP-1 and HIAP-2 expressions than the 25 g/mL geniposide-treated cells, and the lowest expressions were shown by LcS-geniposide-H-treated cells.

### 2.7. mRNA and Protein Expressions of NF-κB and IκB-α in HSC-3 Cells

**Figure 7:**
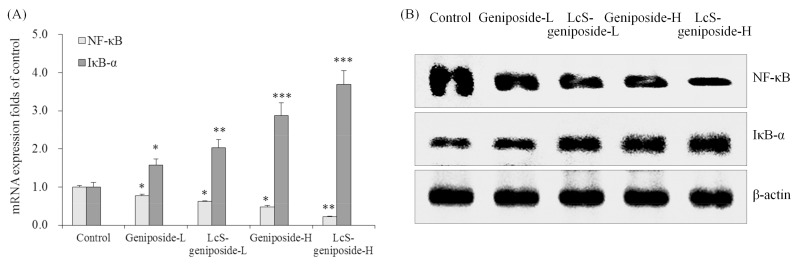
The mRNA (**A**) and protein (**B**) expressions of NF-κB and IκB-αin human oral squamous carcinoma HSC-3 cells. * *p* < 0.05, ** *p* < 0.01, *** *p* < 0.001 vs. the control group. Geniposide-L: 25 µg/mL geniposide; LcS-geniposide-L: 1.0 × 10^6^ CFU/mL LcS + 25 µg/mL geniposide; Geniposide-H: 50 µg/mL geniposide; LcS-geniposide-H: 1.0 × 10^6^ CFU/mL LcS + 50 µg/mL geniposide.

Geniposide could reduce the NF-κB mRNA and protein expressions and raise the IκB-α expressions compared to the untreated control cancer cells (Figure 7). LcS could increase the expressions that increase as an effect of geniposide treatment, and therefore, LcS-geniposide-H-treated cells showed the weskest NF-κB expression and strongest IκB-α expression, which showed that LcS enhances the effects of geniposide.

### 2.8. mRNA and Protein Expressions of Fas and FasL in HSC-3 Cells

**Figure 8:**
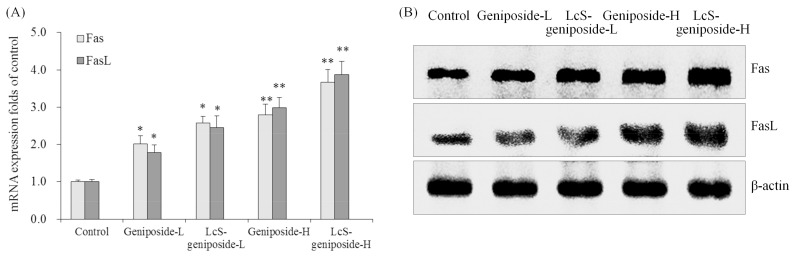
The mRNA (**A**) and protein (**B**) expressions of Fas and FasLin human oral squamous carcinoma HSC-3 cells. * *p* < 0.05, ** *p* < 0.01 vs. the control group. Geniposide-L: 25 µg/mL geniposide; LcS-geniposide-L: 1.0 × 10^6^ CFU/mL LcS + 25 µg/mL geniposide; Geniposide-H: 50 µg/mL geniposide; LcS-geniposide-H: 1.0 × 10^6^ CFU/mL LcS + 50 µg/mL geniposide.

The Fas and FasL mRNA and protein expressions were increased by geniposide treatment, and after the addition of LcS treatment, the Fas and FasL expressions were substantially higher than with only geniposide treatment, with the group having the highest concentration of geniposide and LcS (LcS-geniposide-H)-treated cells showing the highest Fas and FasL expressions (Figure 8).

### 2.9. mRNA and Protein Expressions of TIMP-1, TIMP-2, MMP-2 and MMP-9 in HSC-3 Cells

**Figure 9:**
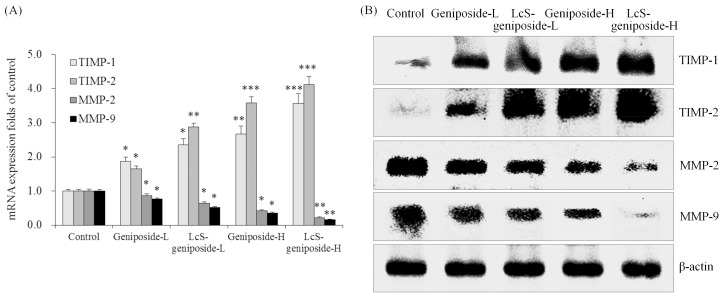
The mRNA (**A**) and protein (**B**) expressions of TIMP-1, TIMP-2, MMP-2 and MMP-9in human oral squamous carcinoma HSC-3 cells. * *p* < 0.05, ** *p* < 0.01, *** *p* < 0.001 vs. the control group. Geniposide-L: 25 µg/mL geniposide; LcS-geniposide-L: 1.0 × 10^6^ CFU/mL LcS + 25 µg/mL geniposide; Geniposide-H: 50 µg/mL geniposide; LcS-geniposide-H: 1.0 × 10^6^ CFU/mL LcS + 50 µg/mL geniposide.

Geniposide-treated HSC-3 cells hadhigher TIMP-1 and TIMP-2 mRNA and protein expressions as well as lower MMP-2 and MMP-9 expressions than untreated HSC-3 cells (Figure 9). The addition of LcS could enhance these changes, with LcS-geniposide-H-treated cells having the highest TIMP-1 and TIMP-2 expressions as well as the lowest MMP-2 and MMP-9 expressions.

### 2.10. mRNA and Protein Expressions of COX-2 and iNOS in HSC-3 Cells

**Figure 10:**
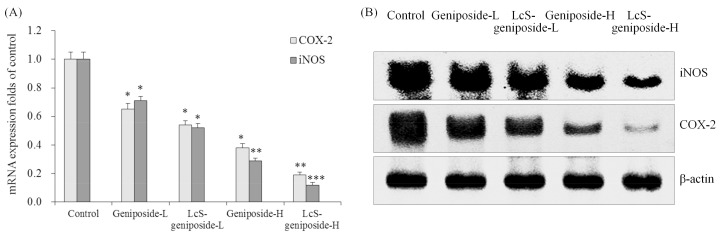
The mRNA (**A**) and protein (**B**) expressions of COX-2 and iNOSin human oral squamous carcinoma HSC-3 cells. * *p* < 0.05, ** *p* < 0.01, *** *p* < 0.001 vs. the control group. Geniposide-L: 25 µg/mL geniposide; LcS-geniposide-L: 1.0 × 10^6^ CFU/mL LcS + 25 µg/mL geniposide; Geniposide-H: 50 µg/mL geniposide; LcS-geniposide-H: 1.0 × 10^6^ CFU/mL LcS + 50 µg/mL geniposide.

The LcS-geniposide-H combination had the weakest COX-2 and iNOS mRNA and protein expressions compared to other groups (Figure 10), and the LcS-geniposide-L-treated cells also showed weaker expressions than the geniposide-L-treated cells.

### 2.11. LcS TransformsGeniposide to Genipin

**Figure 11:**
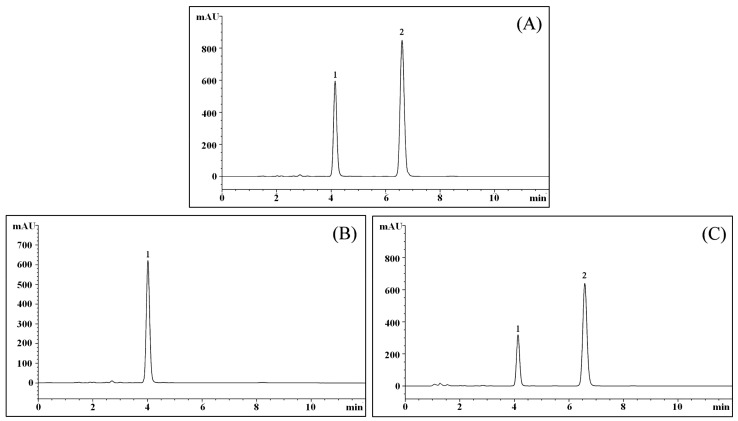
Chromatograms of geniposide and genipin. (**A**) Chromatogram of geniposide and genipin standards; (**B**) Chromatogram of the geniposide solution; (**C**) Chromatogram of LC-Qian-treated geniposide solution; 1: geniposide; 2: genipin.

After evaluating the filtered culture solution, the results showed that geniposide could be transformed to genipin by LcS (Figure 11), and after LcS treatment for 48 h, most of the geniposide-H (Figure 11B) was transformed to genipin (Figure 11C).

### 2.12. Enzyme Activity of LcS Produce β-Glucosidase

The concentration of LcS was adjusted to 1.0 × 10^6^ CFU/mL, after culturing for 48 h, the β-glucosidase was determination, the concentration of LcS produce β-glucosidase was 31.26 ± 3.13 U/L. Under the same cultured condition, LcS were cultured with 25 or 50 µg/mL geniposide, the β-glucosidase concentrations were reduced to 23.18 ± 1.82 and 10.45 ± 1.37 U/L. These results showed that geniposide could consume β-glucosidase, according to the HPLC results could be seen that after β-glucosidase was consumed by geniposide, geniposide was changed to genipin. These enhancements of cancer cell inhibition came from the geniposide hydrolysis translate into genipin through β-glucosidase produced by LcS.

## 3. Discussion

Inducing apoptosis in cancer cells is an important indicator for cancer cell proliferation inhibition treatment [13,14]. Caspase-8 is an upstream protein in an exogenous apoptotic pathway. It can cleave downstream apoptotic executive proteins including caspase-3, 6 and 7, thereby promoting cell apoptosis [15]. The caspase supplementation domain and the caspase-9 precursor binding domain can both lead to caspase-9 self-shearing through Apaf-l. Activated Caspase-9 can activate its downstream Caspases (3, 6 and 7), inducing cell apoptosis from internal pathways. Caspase-3 is the common executive protein of the two apoptotic pathways. Many apoptotic factors ultimately promote cell apoptosis through their common downstream effector, caspase-3 [16,17]. LcS-geniposide could raise caspase-3, 8, and 9 expression in HSC-3 cells, these effects could induce the cancer cells apoptosis.

The inhibition of apoptosis is an important factor in the initiation of cancer. The related proteins of the Bcl-2 family play an important role in the apoptosis regulation of cancer cells. The Bcl-2 family includes apoptosis promoting factors, such as Bcl-2, Bcl-xL and Bax [18]. The ratio of inhibitors of apoptosis to apoptosis promoting factors is related to whether cells receive apoptotic signals. Apoptosis or the inhibition of apoptosis is achieved through the relationship between the two genes and the regulation of each [19]. The dysregulation of apoptosis has an important influence on the development of tumours. Bcl-2 family proteins mainly act through the mitochondrial pathway involved in the regulation of apoptosis, when cells receive dead cell signals. Combined with Bcl-2 or Bcl-xL, Bax is replaced, increasing mitochondrial membrane permeability, and causing the release of a series of apoptosis promoting substances, causing cell death [20]. LcS-geniposide also had the effects of increasing Bax and decreasing Bcl-2, Bcl-xL expression for killing cancer cells, these effects were similar to previous studies.

Fas and FasL are important proteins that mediate apoptosis, and FasL can be induced by the stress response to ultraviolet and DNA damage. At the same time, the interaction of FasL-Fas can spontaneously induce programmed cell death, which is an important mechanism for an organism to scavenging mutant cells [21]. If the expression of FasL in tumour cells is upregulated, tumour-specific antigen can induce tumour infiltrating T lymphocytes to produce high levels of Fas, which makes T cells sensitive to apoptosis. The process of tumour cells inducing high levels of Fas expression in T lymphocytes through their expression of FasL can induce immune suppression. Fas mediated apoptosis is also associated with a variety of factors, including p53 mutation and a lack of CO stimulators [22]. In order to fight cancer, LcS-geniposide could increase the expression of Fas and FasL in HSC-3 cancer cells.

p53 is also a major apoptosis related protein, which can regulate the Bcl-2 family proteins, although p53 has different regulation methods for different proteins in the Bcl-2 family. p53 can increase Bax, it can also down-regulate Bcl-2 or Bcl-xL to promote apoptosis. The permeability of p53, through its interaction with the Bcl-2 family, regulates the cell’s mitochondria [23]. p21, regulating downstream apoptotic genes, is a tumour suppressor gene, and a low concentration of p21 protein can play a role in the aggregation of CDK. This can also promote cell cycle progression by regulating CDK function, promoting G1 to S phase transition. The p21 protein and the high expression of cyclin can competitivly bind with CDK and can inhibit the activity of CDK, leading to a cell cycle arrest at G1 or in S phase, inhibited cell proliferation and induced cell apoptosis [24]. p53 proteins have homology in target gene binding, but their functions are quite different [25]. LcS-geniposide had the cancer cell antiproliferative effect through their p53 and p21 raising effects.

HIAP-1 and HIAP-2 are important apoptosis genes that can inhibit apoptosis induced by caspase proteins, which weakens their effect. Therefore, by controlling the content and weaken HIAP-1 and HIAP-2 in tumour cells, caspases can be fully activated, inducing apoptosis in cancer cells [26]. LcS-geniposide might play the cancer cell antiproliferative effect through regulating the expression of HIAP-1 and HIAP-2 and inducing apoptosis.

NF-kappa B and its inhibitor I kappa B alpha form the NF-kappa B system. NF-kappa B is an extremely important transcription activating factor, and I kappa B alpha is an inhibitor protein of NF-kappa B [27]. NF-kappa B is a key regulatory protein in carcinogenesis. It plays a very important role in the information transmission of the tumour growth process and is closely related to the occurrence and development of tumours [28]. NF-kappa B is highly expressed in a variety of tumours, and the activation of NF-kappa B can promote the change in the expression of a variety of oncogenes [29,30]. The activation of NF-kappa B and the expression of COX-2 can promote the development of cancers [31]. Reducing NF-kappa B and raising I kappa B alpha could inhibit cancer, LcS-geniposide also had these effects, their cancer cell antiproliferative effect might come from the expression changes of NF-kappa B and I kappa B alpha.

COX-2 and iNOS are not only key factors of inflammation, but they also play an important role in the development of tumours. The increased expression of COX-2 and iNOS leads to a change in the signal transduction pathway in cells, and can cause the occurrence, invasion and metastasis of tumours [32]. At the same time, iNOS can induce the high expression of COX-2, thus enhancing the activity of COX-2. Therefore, COX-2 and iNOS are complementary in the promoting the occurrence of tumours. Reducing the expression of COX-2 and iNOS can inhibit the proliferation of tumour cells and promote the apoptosis of cancer cells [33]. Inflammatory expressions are also important expressions related to cancer. LcS-geniposide could also inhibit the cancer cells by affecting the expressions of COX-2 and iNOS of inflammation.

Local invasion and metastasis are important manifestations of the malignancy of cancer cells. In tumour invasion and metastasis, MMPs plays an important role. Not only can MMPs promote degradation mediated by tumour cells of the extracellular matrix host, but they also regulate tumour angiogenesis, cell adhesion molecule function, and can also regulate tumour cell growth. The expression of MMP-2 and MMP-9 of the MMP family is closely related to the formation of neovascularization [34]. High expression of MMP-2 and MMP-9 can enhance the invasion and metastasis ability of tumour cells. Inhibition of the activity of MMP-2 and MMP-9 in tumour cells can inhibit the effects of cancer cells. In addition, the extracellular matrix is a key factor in preventing the invasion and metastasis of cancer cells. There are many factors involved in the degradation of extracellular matrix [35]. MMPs family factor plays a key role in this process, and it can directly degrade the ECM. At the same time, TIMP-1 and TIMP-2 inhibit the degradation of extracellular matrix by inhibiting the activity of MMP-2 and MMP-9 to protect normal cells [36]. The balance of MMPs to TIMPs is related to the formation of vascular endothelium. This can destroy the balance of MMPs-TIMPs, inhibiting angiogenesis, as well as the infiltration and metastasis of tumour cells. By strengthening the expression of TIMPs in the body, tumour invasion and metastasis can be inhibited, and the regulation of TIMPs has become an important part of the study of tumour suppression [37]. LcS-geniposide could also control metastasis related expression of cancer and play an anti-cancer role.

Cytokines have a direct killing effect on tumor cells. A study showed that some beneficial lactic acid bacteria could stimulate the level of related cytokines to inhibit the cancer cells [38]. Lactic acid bacteria could also play a toxic role in cancer cells, thereby prolonging the latent period of cancer [39]. More studies had shown that lactic acid bacteria could inhibit the formation, growth, metastasis and recurrence of Lewis lung cancer, MethA fibroma, B16 melanoma, lymphoma and other kinds of tumor to some extent [40,41]. Genipin is the key cancer cells antiproliferative effect matter, and geniposide could be transformed to genipin by β-glycosidase produced from lactic acid bacteria [42]. LcS is a good probiotic, and it could produce β-glycosidase. As demonstrated in this study, LcS could make geniposide transform to genipin, and the produced genipin increased the antiproliferative effect in oral cancer cells. The LcS in this study may not only have their own anti-cancer effects, but also had the ability to stimulate the anti-cancer substances of geniposide to play a better role. The combination of the two had the better effects.

Lactic acid bacteria and their metabolites can activate the immune system and even induce cancer cell apoptosis, thereby inhibiting the proliferation of cancer cells [43]. In this study, when concentration was less than 10^6^ CFU/mL, LcS had little effect on cancer cells. The β-glucosidase produced by LcS could transform geniposide into genipin, thereby enhancing the inhibitory effect on cancer cells. However, the possibility of a direct antiproliferative effect of LcS alone remains to be studied further. The role of the inactivated LcS and their proliferative state remains to be observed continuously. In particular, the mechanism of β-glucosidase in this course needs be studied in more detail. The effects of geniposide and genipin also need further comparison, especially under in vivo conditions in animals. These further studies may open the possibility to establish a new method for cancer cell inhibition.

## 4. Materials and Methods

### 4.1. Preparation of the Experimental Sample

The sample *Lactobacillus casei* strain Shirota was obtained from the milk probiotic drink Yakult Light (Yakult China Ltd., Shanghai, China). A geniposide standard was obtained from the Shanghai Yuanye Biological Technology Co., Ltd. (Shanghai, China).

### 4.2. Cell Lines

Human oral squamous carcinoma HSC-3 cells were purchased from Shanghai Mingjin Biological Technology Co., Ltd. (Shanghai, China) and HOK normal oral epithelial cells were purchased from ScienCell Research Laboratories (Carlsbad, CA, USA). The HSC-3 and HOK cells were cultured using DMEM medium (Biosera, Nuaille, France) with 10% FBS at 37 °C in a CO_2_ incubator (5%). Furthermore, the DMEM medium was replaced three times a week.

### 4.3. Preparation for the Combination of Geniposide and LcS

The geniposide and LcS were mixed in RPMI1640 medium (geniposide: 25 or 50 μg/mL; LcS: 1 × 10^6^ CFU/mL). Afterwards, the mixture in culture medium was used for the MTT assay.

### 4.4. MTT Assay

HOK or HSC-3 cells were cultured in the dish which contained sterilized DMEM medium, and the concentration of cells was adjusted to 2 × 10^4^/dish. Then the cells solution was added into the 96-well plate with 50 μL per well, the cells were cultured with 5% CO_2_ at 37 °C for 24 h. Sterile geniposide and LcS were added into the DMEM medium to prepare geniposide-L (25 μg/mL), geniposide-H (50 μg/mL), geniposide-L + LcS (1.0 × 10^6^ CFU/mL) and geniposide-H + LcS solutions, then these solutions were added into the 96-well plate with 50 μL per well. In the meantime, 50 μL of culture solution was added to the control group and cultured in a CO_2_ incubator for 48 h. Subsequently, the untreated cells (control group) was added to MTT solution after the removal of clear solution and thereafter incubated for 4 h. In the blank control group, 100 μL of DMSO was added after the removal of the supernatant, followed by shocking for 30 min. The microplate reader was used for detection at 570 nm (680 Microplate Reader, Bio-Rad, Hercules, CA, USA) [13].

### 4.5. Flow Cytometry Assay

The centrifugation of the single cell suspension should be done in order to remove stationary liquid. This was followed by washing with 3 mL of PBS twice, centrifugation for 5 min, the addition of1 mL of PI staining solution and incubation in a refrigerator at 4 °C for 30 min while keeping it in a dark place to prohibit sunlight exposure. The solution was then filtered through a 500-hole copper mesh; flow cytometry detection and an argon ion laser with a 15 mA excitation light source and 488 nm wavelength were used for testing, along with a 630 nm band pass filter to receive the light. The selection of 10,000 cells occurred using the FSC/SSC scattered point diagram method, and we also used gating technology to eliminate adhesive cells and cell debris. This allowed the analysis of the percentage of apoptotic cells in the PI fluorescence histogram [14].

### 4.6. Real-Time Quantitative PCR Assay

The whole RNA of cancer cells was extracted using RNAzol, and RNase-free was used to digest the total RNA for 15 min (37 °C). Then, the RNeasy kit was used to purify the RNA and to adjust the extract to 1 μg/μL. Approximately RNA (2 μg) was used as the template to synthesize cDNA by reacting with reverse transcriptase at 37 °C for 120 min, at 99 °C for 4 min, and at 4 °C for 3 min. Next, the real-time quantitative PCR method was adopted to amplify the gene expression (Table 2) [44] to determine the transcription level of mRNA, and GAPDH was used as the housekeeping gene of the internal control group. The PCR reaction programme was as follows: pre-denaturation at 95 °C for 3 min, denaturation at 95 °C for 10 s, annealing at 57 °C for 30 s, extension at 72 °C for 15 s, and 40 cycles. The mRNA relative levels were determiend using the 2^−ΔΔCr^ formula [15].

### 4.7. Western Blot Assay

After cell culture, simultaneously the protein lysates of cells were combined, and finally, we obtained the total protein extracts. The Bradford method was used to determine the protein concentrations. A separation gel (10%) and stacking gel (5%) were prepared for SDS-PAGE electrophoresis and transmembrane analysis. Every group was isolated for 2 h using non-fat milk sealing liquid (5%), and thereafter, it was combined with primary antibody of caspase-3 (No. PA5-16332, 1:500 dilution, Thermo Fisher Scientific, Waltham, MA, USA), caspase-8 (No. MA5-11558, 1:2000 dilution, Thermo Fisher Scientific), caspase-9 (No. PA5-16355, 1:500 dilution, Thermo Fisher Scientific), Bax (No. MA5-14003, 1:100 dilution, Thermo Fisher Scientific), Bcl-2 (No. MA5-11757, 1:400 dilution, Thermo Fisher Scientific), Bcl-xL (No. MA5-15142, 1:1000 dilution, Thermo Fisher Scientific), Fas (No. ab82419, 1:1000 dilution, Abcam, Cambridge, MA, USA), FasL (No. ab15285, 1:1000 dilution, Abcam), p53 (No. MA5-12557, 1:200 dilution, Thermo Fisher Scientific), p21 (No. MA5-14949, 1:1000 dilution, Thermo Fisher Scientific), HIAP-1 (No. PA1-26473, 1:200 dilution, Thermo Fisher Scientific), HIAP-2 (No. PA5-47036, 1:400 dilution, Thermo Fisher Scientific), NF-κB (No. ab195854, 1:2000 dilution, Abcam), IκB-α (No. ab7217, 1:2000 dilution, Abcam), COX-2 (No. 35-8200, 1:500 dilution, Thermo Fisher Scientific), iNOS (No. PA3-030A, 1:2000 dilution, Thermo Fisher Scientific), MMP-2 (No. 436000, 1:500 dilution, Thermo Fisher Scientific), MMP-9 (No. MA5-15886, 1:1000 dilution, Thermo Fisher Scientific), TIMP-1 (No. MA1-773, 1:500 dilution, Thermo Fisher Scientific), TIMP-2 (No. MA5-12207, 1:200 dilution, Thermo Fisher Scientific) and β-actin (No. MA1-140, 1:500 dilution) at 4 °C for 12 h. Individually, each group was mixed with secondary antibody after being washed with TBST three times and incubated by shaking at 25 °C for 2 h, followed by rinsing with TBST three times. Afterwards, the ECL colouration method was used to make the cells of each group visible, and the GIS gel image was used to analyse the system and for processing [13].

### 4.8. Liquid Chromatography Experiment

Sterilized geniposide and genipin standards (Yuanye Biological Technology Co., Ltd., 2 mg/mL) were prepared in 10 mL volumetric flasks, respectively. The liquid chromatography experiment was performed under the following conditions: C18 chromatographic column (2.1 mm × 50 mm, 1.7 μm, Elite HPLC, Dalian, Liaoning, China); the column temperature should be maintained at 25 °C; the mobile phase was 35–65% methanol-water; the flow rate was 1 mL/min; the detection wavelength was 238 nm; and the volume of the cell treatment was 2 μL (geniposide group, 2 mg/mL; geniposide and LcS mixture group).

### 4.9. β-glucosidase Determination

The culture solution was centrifuged (4000 rpm, 10 min) and the liquid was taken from the upper layer for determination of β-glucosidase used a kit (BC2560, Solarbio, Beijing, China).

### 4.10. Statistical Analysis

The experimental data were expressed using the mean ± standard (SD) deviation. The significant differences (*p* < 0.05, *p* < 0.01 and *p* < 0.001) of data of different groups were calculated using SPSS 12.0 (IBM Corporation, Armonk, NY, USA).

## 5. Conclusions

In this study, the antiproliferative effect of LcS on geniposide in human oral squamous carcinoma HSC-3 cells were determined by MTT assay, flow cytometry, qPCR and western blot experiments. Geniposide showed strong antiproliferative effect in HSC-3 cancer cells, and LcS could increase the antiproliferative effect in a low concentration. From these results, it is understandable that LcS could enhance the antiproliferative effect of geniposide as a sensitizing agent. In conclusion, it will have to be demonstrated in further studies whether this combination could be used effectively in cancer treatment.

## Figures and Tables

**Table 1:** Growth inhibition of human oral squamous carcinoma HSC-3 cells by *Lactobacillus casei* strain Shirota (LcS) and geniposide by an MTT assay.

Treatment	OD_570_ Value	Inhibitory Rate (%)
Control	0.479 ± 0.007	/
LcS	0.454 ± 0.005 *	5.2 ± 0.3
Geniposide-L	0.365 ± 0.010 *	23.8 ± 2.6
LcS-geniposide-L	0.286 ± 0.009 *	40.3 ± 2.3
Geniposide-H	0.212 ± 0.008 **	55.7 ± 2.1
LcS-geniposide-H	0.162 ± 0.005 **	73.7 ± 1.8

* *p* < 0.05, ** *p* < 0.01 vs. the control group. LcS: 1.0 × 10^6^ CFU/mL LcS; Geniposide-L: 25 µg/mL geniposide; LcS-geniposide-L: 1.0 × 10^6^ CFU/mL LcS + 25 µg/mL geniposide; Geniposide-H: 50 µg/mL geniposide; LcS-geniposide-H: 1.0 × 10^6^ CFU/mL LcS + 50 µg/mL geniposide.

**Table 2:** Sequences of primers were used in this study.

Gene Name	Sequence
Caspase-3	Forward: 5′-CAA ACT TTT TCA GAG GGG ATC G-3′
	Reverse: 5′-GCA TAC TGT TTC AGC ATG GCA-3′
Caspase-8	Forward: 5′-CCC CAC CCT CAC TTT GCT-3′
	Reverse: 5′-GGA GGA CCA GGC TCA CTT A-3′
Caspase-9	Forward: 5′-GGC CCT TCC TCG CTT CAT CTC-3′
	Reverse: 5′-GGT CCT TGG GCC TTC CTG GTA T-3′
Bax	Forward: 5′-AAG CTG AGC GAG TGT CTC CGG CG-3′
	Reverse: 5′-CAG ATG CCG GTT CAG GTA CTC AGT C-3′
Bcl-2	Forward: 5′-CTC GTC GCT ACC GTC GTG ACT TGG-3′
	Reverse: 5′-CAG ATG CCG GTT CAG GTA CTC AGT C-3′
Bcl-xL	Forward: 5′-CCC AGA AAG GAT ACA GCT GG-3′
	Reverse: 5′-GCG ATC CGA CTC ACC AAT AC-3′
Fas	Forward: 5′-GAA ATG AAA TCC AAA GCT-3′
	Reverse: 5′-TAA TTT AGA GGC AAA GTG GC-3′
FasL	Forward: 5′-GGA TTG GGC CTG GGG ATG TTT CA-3′
	Reverse: 5′-TTG TGG CTC AGG GGC AGG TTG TTG-3′
p53	Forward: 5′-GCT CTG ACT GTA CCA CCA TCC-3′
	Reverse: 5′-CTC TCG GAA CAT CTC GAA GCG-3′
p21	Forward: 5′-CTC AGA GGA GGC GCC ATG-3′
	Reverse: 5′-GGG CGG ATT AGG GCT TCC-3′
HIAP-1	Forward: 5′-GCC TGA TGC TGG ATA ACT GG-3′
	Reverse: 5′-GGC GAC AGA AAA GTC AAT GG-3′
HIAP-2	Forward: 5′-GCC TGA TGC TGG ATA ACT GG-3′
	Reverse: 5′-GCT CTT GCC AAT TCT GAT GG-3′
NF-κB	Forward: 5′-CAC TTA TGG ACA ACT ATG AGG TCT CTG-3′
	Reverse: 5′-CTG TCT TGT GGA CAA CGC AGT GGA ATT-3′
IκB-α	Forward: 5′-GCT GAA GAA GGA GCG GCT ACT-3′
	Reverse: 5′-TCG TAC TCC TCG TCT TTC ATG GA-3′
COX-2	Forward: 5′-TTA AAA TGA GAT TGT CCG AA-3′
	Reverse: 5′-AGA TCA CCT CTG CCT GAG TA-3′
iNOS	Forward: 5′-AGA GAG ATC GGG TTC ACA-3′
	Reverse: 5′-CAC AGA ACT GAG GGT ACA-3′
MMP-2	Forward: 5′-CTT CTT CAA GGA CCG GTT CA-3′
	Reverse: 5′-GCT GGC TGA GTA CCA GTA-3′
MMP-9	Forward: 5′-TGG GCT ACG TGA CCT ATG AC-3′
	Reverse: 5′-GCC CAG CCC ACC TCC ACT CC-3′
TIMP-1	Forward: 5′-GTC AGT GAG AAG CAA GTC GA-3′
	Reverse: 5′-ATG TTC TTC TCT GTG ACC CA-3′
TIMP-2	Forward: 5′-TGG GGA CAC CAG AAG TCA AC-3′
	Reverse: 5′-TTT TCA GAG CCT TGG AGG AG-3′
GAPDH	Forward: 5′-CGG AGT CAA CGG ATT TGG TC-3′
	Reverse: 5′-AGC CTT CTC CAT GGT CGT GA-3′
